# Illness acceptance and early self-management in older adults with prediabetes: the mediating role of depression

**DOI:** 10.3389/fpubh.2026.1799806

**Published:** 2026-07-03

**Authors:** Huifang Tao, Lihui Yan, Wenqing Guan, Lili Yang, Yuting Yang, YanLing Fang, YuQin Zhou, Jing Xie, Qi Zhou

**Affiliations:** 1 Hangzhou Linping District Hospital of Integrated Traditional Chinese and Western Medicine, Hangzhou, China; 2 Sir Run Run Shaw Hospital, Zhejiang University School of Medicine, Hangzhou, China; 3 Yuhang District Fifth Hospital, Hangzhou, China

**Keywords:** depression, diabetes, older, patient acceptance of health care, self-management

## Abstract

**Background:**

Early self-management is essential for preventing the progression from prediabetes to diabetes, particularly among older adults who often experience difficulties maintaining stable health behaviors during the early adaptation stage. Illness acceptance represents an important cognitive response to disease-related stress, while depression reflects a common emotional reaction during early disease awareness. However, the psychological mechanisms linking illness acceptance and early self-management in older adults with prediabetes remain insufficiently understood.

**Objective:**

This study aimed to examine the relationships among illness acceptance, depression, and early self-management in older adults with prediabetes, and to investigate the mediating role of depression within the stress–cognitive appraisal–coping framework.

**Methods:**

A cross-sectional study was conducted among 400 older adults with prediabetes recruited from multiple hospitals in Zhejiang Province, China, between June and December 2025. Participants completed validated measures assessing illness acceptance (Acceptance of Illness Scale), depressive symptoms (PHQ-3), and self-management behaviors (Self-Management Scale). Pearson correlation analysis was used to assess associations among variables. Mediation analysis was conducted using PROCESS Model 4 with bias-corrected bootstrapping after adjustment for demographic and clinical covariates.

**Results:**

Illness acceptance was positively associated with depressive symptoms (*r* = 0.38, *p* < 0.001) and self-management behaviors (*r* = 0.36, *p* < 0.001). Depressive symptoms were also positively associated with self-management (*r* = 0.67, *p* < 0.001). Mediation analysis demonstrated that depression partially mediated the relationship between illness acceptance and self-management, accounting for approximately 68.57% of the total effect. The indirect effect was statistically significant based on bootstrap confidence intervals. Structural equation modeling indicated acceptable model fit.

**Conclusion:**

Illness acceptance was positively associated with early self-management among older adults with prediabetes, both directly and indirectly through emotional pathways. The findings suggest that emotional responses accompanying early disease awareness may influence behavioral engagement during disease adaptation. Integrating acceptance-based education with emotional support strategies may enhance early self-management interventions for older adults with prediabetes.

## 1 Background

The concept of diabetes self-management was first systematically articulated by the American Diabetes Association (ADA) (1). Diabetes self-management emphasizes patients’ sustained engagement in daily behaviors, including dietary regulation, physical activity, blood glucose monitoring, and adherence to treatment regimens (1). Evidence suggests that self-management behaviors are still in a formative stage during the early phase following diagnosis, with markedly lower stability and coherence compared with the middle and later stages of the disease trajectory (2). Moreover, multiple studies have demonstrated that early self-management is closely associated with subsequent glycemic control, the development of complications, and patterns of healthcare utilization (3, 4). Accordingly, early-stage self-management is regarded as a critical starting point in the long-term management of diabetes, warranting further investigation of its underlying psychological determinants (5).

Acceptance of Illness (AIS) was first proposed by Felton et al. (29) and refers to the extent to which individuals psychologically accept the limitations and changes imposed by disease. Research in individuals with diabetes indicates that illness acceptance is significantly associated with depression, depression, and quality of life (6). Some studies further report that acceptance of illness is generally lower at the time of initial diagnosis than during the stable phase of the disease, a period often characterized by psychological conflict related to long-term treatment demands and lifestyle modification (7, 8). Given that illness acceptance has been shown to influence patients’ engagement in health-related behaviors, its role in the early stage of diabetes merits particular attention (6).

Existing research has largely examined illness acceptance and self-management behaviors as independent constructs in patients with diabetes (6, 9). Studies on illness acceptance have primarily focused on its associations with psychological well-being and quality of life (10), whereas research on self-management has predominantly emphasized its impact on glycemic control and clinical outcomes (3, 4). However, empirical studies directly examining the relationship between illness acceptance and early-stage self-management in diabetes remain limited, and the underlying psychological mechanisms have yet to be fully elucidated. Meanwhile, a growing body of evidence suggests that patients with diabetes commonly experience varying degrees of emotional distress during the early phase of the disease (11, 12). Although prior studies have separately established associations between illness acceptance and depression, as well as between self-management and depression, whether depression mediates the relationship between illness acceptance and early self-management has not been directly tested. This gap constrains a comprehensive understanding of the interplay between psychological adaptation and behavioral management in early diabetes (13).

The stress–cognitive appraisal–coping theory posits that individuals’ responses to stressors depend on their cognitive appraisal of the event and the ensuing emotional reactions (14). In the context of diabetes, the disease constitutes a chronic stressor, while illness acceptance reflects individuals’ cognitive appraisal of disease controllability and coping capacity. From this perspective, illness acceptance shapes emotional responses, particularly depression. Previous studies have consistently shown that lower levels of illness acceptance are significantly associated with higher depression (15). Depression, as a high-arousal negative emotional state, can disrupt attentional allocation, decision-making processes, and behavioral execution, thereby undermining the initiation and maintenance of health-promoting behaviors (16). Accordingly, during the early stage of diabetes, illness acceptance may indirectly influence early self-management behaviors through its impact on depression, with depression serving as a key mediating mechanism linking disease-related cognition to behavioral responses.

The present study aims to systematically examine the relationships among early-stage illness acceptance, depression, and early self-management in patients with diabetes, with a particular focus on testing the mediating role of depression between illness acceptance and early self-management. By addressing the paucity of research on psychological adaptation mechanisms in early diabetes management, this study provides a theoretical basis for integrating assessments of illness acceptance and emotional management into self-management interventions, thereby supporting the development of more targeted and comprehensive early-stage intervention strategies in clinical practice.

## 2 Methods

### 2.1 Participants

This cross-sectional quantitative study used convenience sampling to recruit older adults with diabetes from hospitals at different levels in Zhejiang Province, China. Participants were drawn from general medical departments and diabetes-related specialty clinics, including endocrinology units, to improve sample diversity. The collection period was from June 2025 to December 2025.

Inclusion criteria were: (1) a clinical diagnosis of prediabetes based on established criteria (e.g., impaired fasting glucose or impaired glucose tolerance); (2) adults aged 55 years or older who received a first-time diagnosis of prediabetes and had no prior diagnosis of diabetes; (3) no prior diagnosis of diabetes; and (4) ability to complete the questionnaire independently or with minimal assistance. Participants with a documented diagnosis of major depressive disorder, severe psychiatric illness, cognitive impairment, or current use of psychiatric medication prior to the diagnosis of prediabetes were excluded.

Based on the 10-fold rule for structural equation modeling (SEM) (17, 18), the minimum required sample size was estimated at 420. Mediation analysis using ordinary least squares regression (PROCESS Model 4) was additionally powered to detect small-to-moderate indirect effects using bias-corrected bootstrapping with multiple covariates. A total of 400 valid questionnaires were included in the final analysis, satisfying recommended sample size requirements for both SEM and mediation analyses.

### 2.2 Measures

The survey included standardized scales to measure AIS, SMS and PHQ. It also collected demographic information, including CRP, LDL-C, TG, ALT, AST, HbA1c, Albumin, Gender, Notion, Marriage, Residence, Education, Drinking, Smoking, Excise, Diabetes, Pre-education, and Payment.

Depression. We used Perlis’s 3-item PHQ-3 scale to measure depression (12). The scale used a Likert 5-point scale with a total score range of 3–15, with higher scores indicating higher levels of depression. PHQ-3 scores may be interpreted as reflecting relatively low (3–5), mild-to-moderate (6–9), and higher (10–15) levels of depressive symptoms, with higher scores indicating greater emotional distress. Feeling nervous, anxious, or anxious. The Cronbach *α* of PHQ-3 was 0.88, and the Pearson correlation coefficient between PHQ-3 and the total score of PHQ--9 was 0.93, which suggested that PHQ-3 could well reflect the overall severity of depression.

Acceptance of Illness (AIS). We used Zhao Wenwen’s 8-item Acceptance of Illness Scale to measure Cognitive Control and Flexibility (19). The scale uses a Likert scale with a total score of 8–40, with higher scores indicating higher levels of Acceptance of Illness. I felt that my illness was limiting my daily life. The KMO value of the Chinese version of AIS-CHI was 0.611, and the value of Bartlett’s spherical test was 167.992(*p* < 0.001). Three common factors were obtained by exploratory factor analysis, and the cumulative explanatory variance was 64.36%. The Cronbach’s alpha coefficient of internal consistency of AIS-CHI was 0.754.

SMS. We used Wang Jingxuan’s 29-item self-management Scale to measure prediabetic self-management (1). The self-management scale was adapted to reflect prediabetes-related preventive behaviors, including diet, exercise, and risk awareness. The scale was divided into nine dimensions: health concept, self-efficacy, diet management, exercise management, rest and sleep management, stress coping, compliance management, family environment management, and Social Environment Management. Although the scale includes multiple behavioral dimensions, the total score was used in the present study to reflect overall prediabetes self-management capacity and to maintain consistency with the mediation framework. The scale used Likert 5-point scoring method, and the total score of the scale ranged from 29 to 145. The higher the score, the higher the level of self-management in prediabetes. One sample item is unhealthy eating habits are a risk factor for diabetes. The Chinese version of Cronbach’s alpha is 0.87, and the construct validity is 0.68.

### 2.3 Statistical analysis

Data analyses were performed using SPSS Version 26.0. Descriptive statistics summarized participants’ demographic and clinical characteristics, and Pearson correlation coefficients assessed associations among study variables.

Assumptions for parametric testing were examined prior to hypothesis testing. Skewness (−0.28 to 0.39) and kurtosis (−0.58 to 0.68) indicated approximate normality. Multicollinearity was assessed using variance inflation factors (VIF < 1.8). Common method bias was evaluated using Harman’s single-factor test, with the first factor accounting for 36.9% of the total variance. Mediation analyses were conducted using PROCESS Macro Model 4 by Imai et al. (20). Illness acceptance (AIS) was specified as the independent variable, early self-management (SMS) as the dependent variable, and depression (PHQ-3) as the mediator. Indirect effects were estimated using bias-corrected bootstrapping with 4,000 resamples. Indirect effects were estimated using bias-corrected bootstrapping with 4,000 resamples. Mediation was considered statistically significant when the 95% confidence interval did not include zero. Sobel–Goodman tests were applied as robustness checks (*p* < 0.01).

Structural equation modeling was conducted to evaluate the hypothesized mediation model linking illness acceptance, depression, and self-management. Model fit indices indicated acceptable fit (SRMR = 0.058, CFI = 0.96, RMSEA = 0.055).

### 2.4 Ethics statement

This study was approved by the Ethics Committee of Hangzhou Linping District Integrated Traditional Chinese and Western Medicine Hospital (Approval No. 2024-L-2024-Z-51). All procedures complied with the Declaration of Helsinki and adhered to the STROBE reporting guidelines. Participation was voluntary, informed consent was obtained from all participants, and all data were collected anonymously to protect confidentiality.

## 3 Results

### 3.1 Descriptive statistics

A total of 400 participants were analyzed. Men accounted for 52.5% of the sample, and 47.5% were women; nearly all participants were of Han ethnicity (99.8%). The majority were married (86.5%) and lived with family members (89.8%). Educational levels varied, with 36.3% having primary education or below, 34.8% completing middle school, and 29.0% attaining high school education or higher. Most participants resided in urban areas (77.3%).

Clinical variables were examined for outliers and distributional abnormalities prior to analysis. Extreme values were assessed and verified against original records. All biochemical indicators are reported with standardized units. Clinical assessments showed mean concentrations of CRP, LDL-C, and triglycerides of 11.83 ± 29.82, 5.39 ± 52.67, and 1.75 ± 2.46, respectively. Mean ALT and AST values were 39.23 ± 114.32 and 41.75 ± 118.67, indicating variability in liver function. The average HbA1c level was 5.97 ± 0.22%. Mean albumin and hemoglobin levels were 42.29 ± 7.31 and 134.37 ± 22.83, respectively. Regarding health-related behaviors, most participants reported no alcohol use (70.0%), whereas 27.8% reported current drinking. Smoking prevalence was 18.3%. Physical activity was reported as occasional by 77.8% of participants, while 13.8% engaged in regular exercise (Table 1).

**Table 1:** Characteristics of the patients.

Variables	Mean	SD
CRP	11.83	29.82
LDL-C	5.39	52.67
TG	1.75	2.46
ALT	39.23	114.32
AST	41.75	118.67
HbA1c	6.97	6.22
Albumin	42.29	7.31
Glycated hemoglobin	2.39	6.43
Hemoglobin	134.37	22.83
Gender	Frequency	Percentage
Female	190	47.5
Male	210	52.5
Notion		
Han Zu	399	99.8
Man Zu	1	0.3
Marriage		
Unmarried	34	8.5
Married	346	86.5
Widowed or divorced	20	5
Residence		
Alone	29	7.2
Family	359	89.8
Other	12	3
Education		
Primary and below	145	36.3
Middle School	139	34.8
High School and above	116	29
Drinking		
Not	280	70
Drinking	111	27.8
Stopping Drinking	9	2.3
Smoking		
Yes	73	18.3
No	327	81.8
Excise		
Never	34	8.5
Sometimes	311	77.8
Regularly	55	13.8
Diabetes		
None	327	81.8
Yes	73	18.3
Pre-edu		
No	128	32
Yes	272	68
Pay		
Rural	81	20.3
Urban	309	77.3
Self	2	0.5
Other	8	2

### 3.2 Descriptive statistics and correlation results

The correlation results are shown in Table 2. The score of AIS was 17.82 (SD = 15.25). The PHQ was 3.57 (SD = 1.35). The SMS was 40.74 (SD = 15.04). AIS was significantly positively correlated with PHQ (r = 0.38, *p* < 0.001) and SMS (*r* = 0.36, *p* < 0.01). PHQ was significantly positively correlated with SMS (*r* = 0.38, *p* < 0.001).

**Table 2:** Correlation results.

Variable	AIS	PHQ	SMS
AIS	1	0.38**	0.36**
PHQ	0.38**	1	0.67**
SMS	0.36**	0.67**	1

Significance as follows: **p < 0.001.

### 3.3 Hypothesis testing for pathways

**Figure 1:**
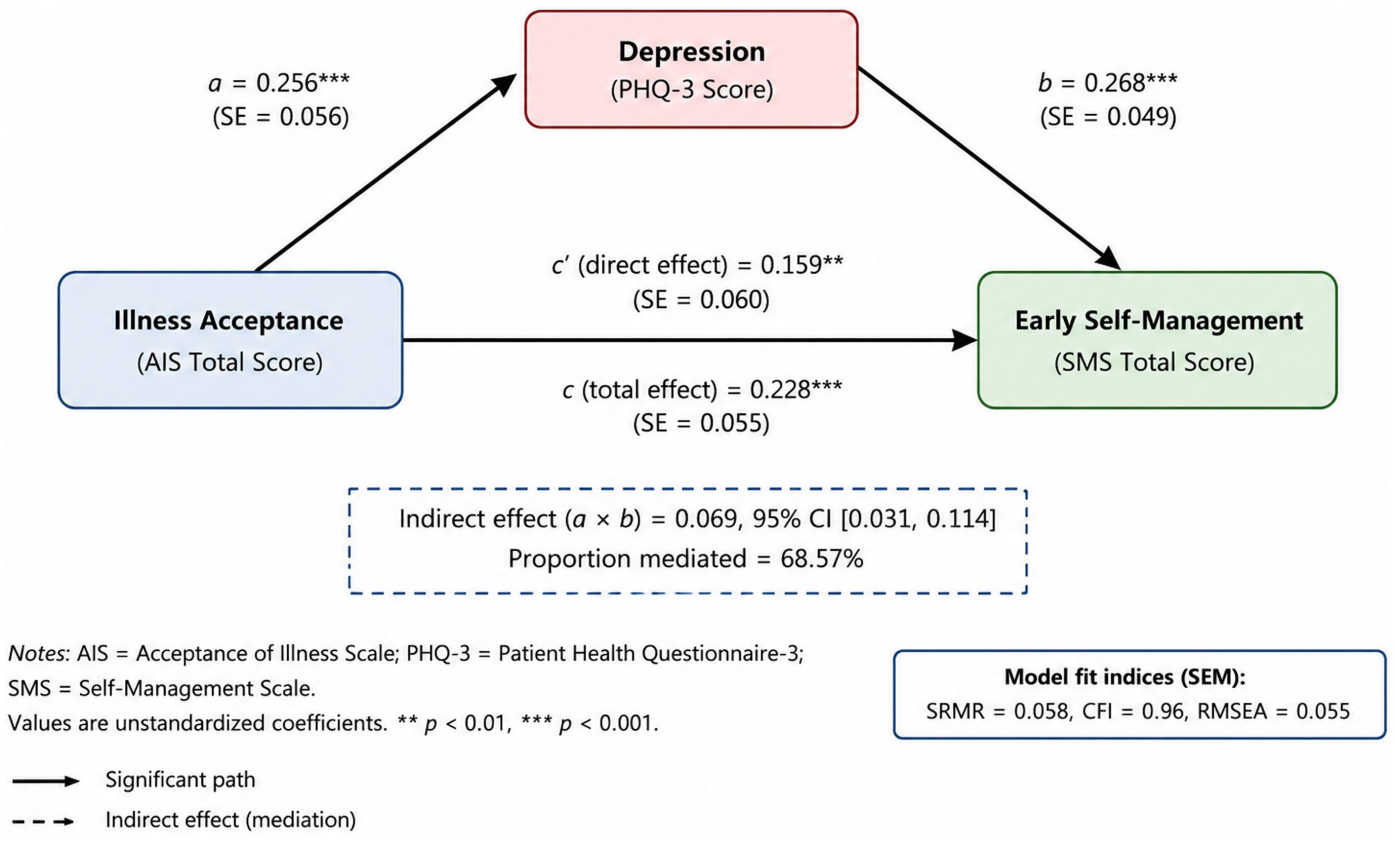
Mediation model of depression in the relationship between illness acceptance and early self-management among older adults with prediabetes.

The PHQ confirmed the relationship between AIS and SMS (Figure 1). AI affected PHQ (*B* = 0.03, *p* < 0.001), with an explained variation of 35%. AIS and PHQ affected SMS (*B*  = 0.11 and 7.01, *p* < 0.001), with an explained variance of 46%. PHQ’s total, direct, and indirect effects were 0.35, 0.11, and 0.24, respectively. All values were significant, indicating that PHQ partially mediated between AIS and SMS (Table 3). The proportion mediated (indirect/total) was approximately 68.57% (0.24/0.35), indicating that a substantial share of the AIS and SMS association operates through PHQ.

**Table 3:** Mediation effect of PHQ between AIS and SMS.

Predictor	PHQ				SMS			
	*B*	SE	*t*	*P*	*B*	SE	*t*	*P*
AIS	0.03	0.01	31.05	<0.001	0.11	0.04	2.93	<0.001
PHQ					7.01	0.44	15.74	<0.001
Adjust *R*^2^	0.14				0.46			
*F*	69.81				171.77			

AIS → PHQ → SMS	Effect	BootSE	95% CI	
			Low bound	Upper bound
Indirect Effect	0.24	0.05	0.41	0.69
Direct Effect	0.11	0.04	0.02	0.22
Total Effect	0.35	0.05	0.26	0.45

## 4 Discussion

This study examined the relationships among illness acceptance, depression, and early self-management in older adults with prediabetes within the stress–cognitive appraisal–coping framework. The findings demonstrated that illness acceptance was positively associated with self-management behaviors both directly and indirectly through depression. These results suggest that psychological adaptation and emotional responses may jointly influence behavioral engagement during the early stage of prediabetes management among older adults.

The present study found that higher illness acceptance was associated with higher levels of depressive symptoms. This finding may initially appear inconsistent with studies in patients with established diabetes, where illness acceptance is often associated with lower psychological distress (8, 15). However, differences in disease stage and population characteristics may partially explain this discrepancy. Prediabetes represents an early and uncertain phase of chronic disease development, during which individuals may become increasingly aware of future health risks and the possibility of progression to diabetes. For older adults, this awareness may heighten concerns regarding physical decline, long-term treatment burden, and loss of independence (21, 22). Within the stress–cognitive appraisal–coping framework, illness acceptance reflects a cognitive acknowledgment of disease-related threats and health vulnerability. During the initial adaptation stage, greater acceptance may therefore temporarily coexist with increased emotional distress, particularly among older individuals with heightened sensitivity to health-related uncertainty.

Importantly, the findings should not be interpreted as suggesting that depression is beneficial or desirable. Rather, the observed association may reflect mild emotional distress accompanying increased disease awareness during the early adaptation process. Existing literature indicates that emotional responses to chronic disease are dynamic and context dependent (11, 13). While severe or persistent depressive symptoms are consistently associated with poorer self-care, reduced motivation, and impaired health outcomes (23), mild disease-related emotional distress may sometimes increase vigilance and perceived urgency regarding health behaviors during the early stages of illness recognition (24). Therefore, the current findings may reflect an adaptive awareness process rather than a protective role of depression itself.

The mediation analysis further demonstrated that depression partially mediated the relationship between illness acceptance and self-management. This finding supports the theoretical assumption that cognitive appraisal influences behavioral responses partly through emotional pathways. Older adults who increasingly recognize the health implications of prediabetes may experience greater emotional arousal, which in turn could motivate engagement in preventive behaviors such as dietary modification, exercise participation, and lifestyle regulation. Nevertheless, this interpretation should be approached cautiously. The positive association observed in this study may be specific to relatively mild depressive symptoms within a prediabetes population and should not be generalized to clinically significant depression. Previous research has predominantly reported negative associations between depression and diabetes self-management, particularly when depressive symptoms become severe or chronic (11, 23). Accordingly, emotional distress in the present study may function more accurately as a transient salience signal that increases health awareness during early disease adaptation rather than as a uniformly adaptive factor. It is also important to consider the measurement approach used in the present study. Depression was assessed using the PHQ-3, a recently developed ultra-short screening instrument that was selected to reduce respondent burden and improve feasibility among older adults participating in a multi-site survey. Previous validation research has demonstrated strong agreement between PHQ-3 and PHQ-9 scores. Nevertheless, because the PHQ-3 contains fewer items and focuses on core depressive symptoms, it may provide a less comprehensive assessment of depression than longer instruments such as the PHQ-9. Therefore, the observed findings should be interpreted as reflecting depressive symptom severity rather than a full clinical evaluation of depression.

Another important finding was that illness acceptance remained directly associated with self-management even after accounting for depression. This suggests that illness acceptance may independently facilitate behavioral engagement beyond its emotional effects. Higher acceptance may reduce denial, increase perceived responsibility for health, and promote readiness to adopt lifestyle modifications (6, 9). For older adults, acceptance of prediabetes may also support the integration of self-management behaviors into daily routines and strengthen long-term adherence to preventive strategies. This finding is consistent with previous studies demonstrating that acceptance-based cognitive processes are positively associated with self-care behaviors and treatment adherence in chronic illness populations (25).

The present findings have several clinical implications. First, psychological adaptation should be considered an important component of early prediabetes management among older adults (26). Healthcare professionals should recognize that emotional distress may emerge during the process of disease acceptance and should provide timely emotional assessment and support. Second, interventions should focus on promoting adaptive emotional regulation and coping rather than implying that depressive symptoms themselves are beneficial (27). Acceptance-based education, supportive counseling, and early psychological guidance may help older adults process disease-related concerns constructively while maintaining engagement in preventive behaviors. Third, integrating psychological support with behavioral self-management programs may improve the effectiveness of early diabetes prevention interventions in aging populations (28).

Several limitations should be acknowledged. First, the cross-sectional design prevents causal inference regarding the temporal relationships among illness acceptance, depression, and self-management. Longitudinal studies are needed to examine whether emotional distress changes over time during disease adaptation. Second, all variables were measured using self-reported questionnaires, which may introduce recall bias and social desirability bias. Third, the sample was limited to older adults with prediabetes recruited from hospitals in one province of China, which may limit generalizability to other populations or healthcare settings. Fourth, depressive symptoms were assessed using the PHQ-3, an ultra-short screening measure selected to reduce participant burden and improve response feasibility among older adults. Although previous validation studies have demonstrated strong correspondence between PHQ-3 and PHQ-9 scores, the PHQ-3 does not capture the full range of depressive symptom domains assessed by longer instruments. Consequently, some aspects of depression severity may have been underrepresented, and the findings should be interpreted as reflecting depressive symptom burden rather than clinically diagnosed depression. Finally, only depression was examined as a mediator, whereas other psychological factors such as anxiety, perceived risk, health literacy, or coping style may also influence early self-management behaviors.

## 5 Conclusion

This study demonstrates that disease acceptance is positively associated with prediabetes self-management in older adults, with depression acting as a partial mediator. These findings underscore the importance of considering emotional responses when promoting self-management behaviors. Integrating acceptance-based education with depression-focused support may enhance the effectiveness of prediabetes interventions in older populations.

## Data Availability

The raw data supporting the conclusions of this article will be made available by the authors, without undue reservation.
